# P2X7Rs: new therapeutic targets for osteoporosis

**DOI:** 10.1007/s11302-021-09836-0

**Published:** 2022-02-02

**Authors:** Haoyun Huang, Yu-Mei He, Miao-Miao Lin, Yanchao Wang, Xiaomei Zhang, Li Liang, Xueling He

**Affiliations:** 1 Clinical Medical School, Chengdu University of Traditional Chinese Medicine, Chengdu, 610072 China; 2 School of Sports Medicine and Health, Chengdu Sports University, Chengdu, 610041 China; 3 Department of Neurosurgery, West China Hospital, Sichuan University, Chengdu, 610041 China; 4 Laboratory Animal Center of Sichuan University, Chengdu, 610041 China; 5 Institute of Biomedical Engineering, West China School of Basic Medical Sciences and Forensic Medicine, Sichuan University, Chengdu, 610041 China

**Keywords:** Inflammation, Osteoporosis, P2X7 receptor, Osteoclasts, Osteoblasts

## Abstract

Increasing evidence suggests that both the occurrence and progression of osteoporosis are associated with inflammation, especially in primary osteoporosis. The maintenance of skeletal homeostasis is dependent on the complex regulation of bone metabolism. Numerous evidence suggested that purinoceptor networks are essential for bone homeostasis. In this review, the relationship between inflammation and the development of osteoporosis and the role of P2X7 receptor (P2X7R) in regulating the dynamic regulation of bone reconstruction were covered. We also discussed how P2X7R regulates the balance between resorption and bone formation by osteoblasts and reviewed the relevance of P2X7R polymorphisms in skeletal physiology. Finally, we analyzed potential targets of P2X7R for osteoporosis.

## Introduction

Bone mass is gradually lost with age, leading to osteoporosis, which is a systemic skeletal disorder characterized by reduced bone mass, increased bone fragility, and fracture risk due to the destruction of bone microstructures [1]. It is estimated that 25% women over 50 years and 20% men over 50 years suffer from osteoporosis. Fractures caused by age-related bone loss are the main cause of disability and death in elderly patients, seriously affecting the lives of the elderly, and bringing a heavy economic burden to patients and society [2, 3]. With the aging of the global population, aging-related osteoporosis has grown up to be a public health problem worldwide.

Osteoporosis is divided into primary osteoporosis (such as postmenopausal osteoporosis and senile osteoporosis) and secondary osteoporosis(such as disease processes. It is now believed that the decline in estrogen levels and bone degenerative changes are the main causes of osteoporosis, but osteoporosis is a complex multi-factor disease involving genetic and environmental factors [4]. In recent years, clinical studies have found that many inflammatory diseases are commonly accompanied by obvious local or systemic bone loss, such as glucocorticoids [5], rheumatoid arthritis [6], diabetes [7], kidney disease [8], cardiovascular disease [9], and so on. These findings suggest that inflammation is closely linked to osteoporosis. With more studies carried out on animals and humans, the evidence that inflammation plays an important role in the occurrence and progression of postmenopausal osteoporosis and age-related osteoporosis is becoming clearer.

In recent years, the role of adenosine triphosphate (ATP) and its associated receptors in the inflammatory process has been identified. ATP is a natural ligand for the P2X receptor (the metabotropic receptor family) and its expression on all osteoblast types (osteoclasts, osteoblasts, and osteocytes) has been demonstrated [10]. There is growing evidence suggesting that purinergic signaling also plays a critical role in the physiological regulation of bone metabolism [11–13]. Although several reviews on the role of P2X receptors in bone biology have been reported, the majority of studies were focused on P2X7R. Activation of P2X7R may ameliorate osteoporosis by regulating the balance between osteoblasts and osteoclasts, and the balance between resorption and bone formation by osteoblasts remains essential for skeletal homeostasis [14, 15]. Therefore, P2X7R has great potential therapeutic targets for inflammatory osteoporosis. In this paper, we systematically reviewed the role of P2X7R in the dynamic regulation of bone reconstruction and its targeting potential.

## Relationship between inflammation and osteoporosis

### Inflammation and the postmenopausal osteoporosis

Decreased ovarian function and decreased estrogen levels in postmenopausal women are the main reasons for rapid bone loss leading to osteoporosis [16]. However, numerous studies have demonstrated that the decline of postmenopausal estrogen levels can increase the inflammatory cytokines [17–19]. Early research has shown that serum inflammatory indexes and P2X7R [20, 21] are significantly higher in women with postmenopausal osteoporosis (PMOP). The T cell activity and nuclear transcription factor-κB receptor activator ligand (RANKL) expression increased, as well as the various inflammatory cytokines released by T cells that promote bone resorption. However, the levels of these inflammatory cytokines reduced significantly when given estrogen replacement therapy [22]. In addition, early clinical research has aslo found that the interleukin-1 (IL-1) and tumor necrosis factor-α (TNF-α) levels in serum were significantly increased in women who had undergone surgical removal of the ovaries, and reduced to preoperative levels after 4 weeks of estrogen replacement therapy [23]. Similar research results were also found in animal studies. The number of thymus, T cells, and the TNF-α released by T cells were significantly increased in ovariectomized mice or rats, and the bone mass can be close to the pre-ovariectomized level after administration of IL-1 receptor antagonist and TNF-binding protein, and the therapeutic effect is similar to estrogen replacement treatment [24–26]. Further research found that the bone loss of ovariectomized TNF^−/−^ mice was considerably less than that of wild-type mice. At the molecular level, TNF-α and IL-1β are considered to be effective inflammatory signals, as well as regulators of osteoclast formation and activity, which may be partly responsible for the rapid premenopausal bone loss [27, 28]. These studies suggested that inflammatory cytokines are essential in the occurrence and development of women with PMOP.

### Inflamma-aging and the senile osteoporosis

Inflamma-aging is a progressive chronic pro-inflammatory state that appears with aging [29]. It plays key roles in many senile diseases, such as Alzheimer, Parkinson, multiple sclerosis, atherosclerosis, diabetes, osteoporosis, and so on [30, 31]. The mechanisms of inflammatory aging are associated with inflammatory cytokines, autophagy, oxidative stress, and DNA damage [32].

Studies have shown that aging is a key driver of bone loss and bone fragility. The bone loss that occurs with aging reflects the confluence of various molecular and cellular processes. Therefore, similar to the mechanism of PMOP, inflammation also plays an important part in senile osteoporosis. The expression levels of inflammatory cytokines such as TNF-α, IL-1, IL-6, and IL-17, which are serum markers of inflammatory-aging, increased with age [33]. Moreover, the increased levels of these inflammatory cytokines produce a large number of osteoclasts and inhibit the activity of osteoblasts [34]. Macrophage colony stimulating factor (M-CSF), which plays an important role in the survival and proliferation of osteoclasts, is an important cytokine during the differentiation of osteoclast precursor cells into mature osteoclasts. TNF-α can promote the production of M-CSF in a direct or indirect manner. In addition, TNF-α can also inhibit the production of osteoblasts, reduce bone matrix calcification, and ultimately lead to an imbalance of bone formation and bone resorption. IL-1 is another important cytokine that affects bone metabolism and bone remodeling activities in the bone microenvironment. It can stimulate osteoclast production and produce a strong effect on bone resorption. Similarly, increased levels of IL-6 can also lead to increasing levels of TNF-α and IL-1. These inflammatory cytokines promote bone resorption by enhancing the activation and differentiation of osteoclasts, and inhibiting the survival of osteoblasts.

Autophagy is the self-protection mechanism of cells, which plays an important role in the maintenance of cellular homeostasis, proliferation, differentiation, and stress. The autophagy level of bone cells gradually decreased with aging, which increased the secretion of pro-inflammatory cytokines, accelerated bone loss, and then leaded to osteoporosis [35]. The ability of cells to be eliminated through autophagy was reduced, and mitochondrial dysfunction leaded to protein accumulation with aging [36]. As a result, oxidative stress and ROS increase, and inflammation occurs, which further lead to increased inflammation and accelerated aging [37].

A recent study found that changes in aging skeletal stem cells (SSC) may be one of the key factors leading to poor fracture healing, osteoporosis, and various blood diseases [38, 39]. Aged bone stem cells have lower activity and poorer ability to form bones. Further research found that the genes expressed by aged bone stem cells are mainly related to decreased bone formation and enhanced bone resorption. Aged skeletal stem cells affected not only the lower ability to form bones, but also the growth of hematopoietic stem/progenitor cells (HSPC), leading to more bone-absorbing cells and producing inflammatory cytokines that cause fibrous tissue instead of bone growth. This imbalance between bone formation and bone resorption ultimately leads to osteoporosis [40].

However, there are still few studies on the related mechanisms of inflamma-aging and osteoporosis. We believe that there will be more studies in the future to explore the impact of the relationship between them on bone metabolism.

## P2X7 receptor and ATP-mediated purinergic signaling during inflammation

ATP exists in all cells and has been identified as an extracellular signaling molecule, which is involved in several signal pathways in various kinds of cells and diseases. ATP is mainly released by immune cells and hematopoietic cells as well as osteoblasts and osteocytes [8,26,]. Compared with cytoplasm, the normal ATP concentration is relatively low in the extracellular, but the extracellular concentration of ATP is usually increased in damaged tissue, tumor, inflammation, or fracture [37, 41]. P2X7R, one of the seven receptor subtypes, plays a key role in mediating the activation of NLRP3 inflammasomes. The non-selective ligand cation channel formed by the activated P2X7R causes lots of Ca^2+^ and Na^+^ influx and changes the membrane potential and leads to the efflux of K^+^ through the TWIK2 (the Kcnk6 gene code) channel. TWIK2 and P2X7R synergistically activate NLRP3 inflammasomes. Activated NLRP3 inflammasomes mediate the activation of caspase-1 to mature the precursor IL-1β and induce the release of mature IL-1β to the cytoplasm. In addition, a large amount of Ca^2+^ influx caused by activated P2X7R can activate calmodulin-dependent protein kinase II and Ca2 + -dependent phospholipase A2, and induce the release of IL-1β [28, 42]. P2X7R can also promote the production of reactive oxygen species (ROS) by macrophages, which is involved in the activation of the p38 and JNK pathways mediated by the nucleotide receptor. However, the activated ROS, p38, and JNK pathways all together play a critical role in a variety of immune response processes [43]. It can be seen that ATP-P2X7R signaling plays an important role in the inflammatory process caused by macrophages.

Beyond these, P2X7R is also involved in the activation and expression of transcription factors, such as nuclear factor-κB (NF-κB). NF-κB is a nuclear transcription factor that controls the expression of a variety of inflammatory genes, including TNF-α, COX-2, and IL-1b, increased TNF-α and IL-1β can enhance NF-κB activation and further exacerbate inflammation in turn. An increasing number of studies link the pro-inflammatory activity of P2X7R to NF-KB nuclear translocation, further confirming the P2X7R-dependent activation of NF-KB in microglia, osteoclasts, and osteoblasts [28, 42].

## Dynamic regulation of P2X7R on bone remodeling

P2X7R, as a ligand ion gating system, is widely distributed in osteocytes, osteoclasts, and osteoblasts, which plays an important role in bone remodelling. Moreover, as a repair receptor, P2X7R can promote the repair and calcification of small fractures, thereby accelerating bone remodeling [44]. A proper balance between osteoclasts and osteoblasts is essential for healthy bones [45]. P2X7R may ameliorate osteoporosis by maintaining a balance between osteoclast and osteoblast activity [46–48].

### The effect of ATP and P2X7R on osteoclasts

#### Osteoclast precursor cells

Osteoclasts, the bone-resorbing cells, are generated from mononuclear monocyte-macrophage precursors that derived from hematopoietic stem cells (HSCs) in the bone marrow. Cells of HSCs lines have also been demonstrated to express multiple purine receptors [49, 50]. Extracellular ATP can promote the transformation of HSCs to myeloid progenitor/osteoclast precursor cells. Studies have shown that a high concentration of ATP (1 mM) can reduce the number of HSCs in mice, while increased in bone marrow cells. HSCs have not changed significantly when exposed to low concentrations of ATP (less than 1 mM) [51]. These indicated that the role of P2X7R in promoting HSCs may be along the osteoclast cell line.

Osteoclasts are large multinucleated terminally differentiated cells formed by the fusion of mononuclear hematopoietic precursors, but the fusion of precursor cells to multinucleated osteoblasts is a complex biological behavior that is not yet completely understood. Recently, it has been shown that osteoclast precursor fusion to form multinucleated cells was significantly inhibited by anti-P2X7R antibodies or antagonists of oxidized ATP, for example, human blood monocyte formation of osteoclast-like cells can be prevented by some P2X7R antagonists in vitro [52, 53]. ATP can also act on P2X7R promoting the fusion and the differentiation of osteoclast precursors, as well as cell apoptosis through downstream signaling pathway, such as PKC translocation, nuclear localization of NF-κB, and activation of nuclear factor of activated T cells 1 (NFATc1) [54]. Beside that, extensive internalization of P2X7R induced by prolonged exposure to ATP can also block the ability of RAW 264.7 cells to fuse into multinucleated osteoclast-like cells [55].

#### Mature osteoclasts

P2X7R is expressed in osteoclasts generated from rodents and rabbits in vitro, and differentiation of primary mouse osteoclasts is dependent on P2X7R expression [56, 57]. P2X7R expression is also present in human monocyte precursors and throughout osteoclastogenesis in vitro, and the expression of mRNA and protein of P2X7R was higher in mature resorbing cells compared to their precursors [53, 58]. Highly expressed R2X7 promotes spontaneous fusion of osteoclasts in vitro. Subsequent studies verified that the addition and accumulation of ATP promoted osteoclast fusion [59]. Therefore, we hypothesized that osteoclast fusion requires the release of ATP through the P2X7R pore, although this action may indirectly involve other purinergic receptors. Interestingly, similar to the HSCs, the ATP-mediated P2X7R also has multiple effects on osteoclasts. ATP could enhance osteoclast formation and resorption when it is at a low concentration (0.2–2 μM). However, higher concentrations (20–200 μM) of ATP may have an adverse effect on osteoblasts, such as the formation of lytic pores that leads to apoptosis and persistent inflammation [60]. It has been noted that bone resorption was decreased by extracellular ATP, which was likely resulted from cytotoxic effect by activated P2X7R on osteoclasts [61]. In summary, activation of P2X7R in osteoclasts is essential for cell fusion and is critical in determining cell survival time and uptake. It can be hypothesized that the formation of osteoclasts and their P2X7R functions are regulated by regulating the extracellular ATP concentration.

### The effect of ATP and P2X7R on osteoblasts

#### Osteogenic precursors cells

Osteoblasts are derived from bone marrow mesenchymal stem cells (BMSCs) and various purinergic receptors which plays an important role in determining the differentiation fate of MSCs. Intracellular ATP releases activate P2X7R and drive osteogenic differentiation of MSCs [62, 63]. That shock wave induces osteogenic differentiation of human MSCs through ATP release and activation of P2X7R had just demonstrated. The researchers found that shock waves caused ATP to be released from hMSCs and led to downstream activation of the 38 MAPK signaling pathway, transcription of c-Fos and c-Jun mRNAs, and osteogenic differentiation [64]. These downstream events were completely abolished when treated with apyrase (an enzyme that hydrolyzes extracellular ATP), P2X7R-siRNA, PPADS (a nonselective P2 antagonist), and KN-62 (a P2X7R antagonist), which suggests P2X7R-mediated these events [64].

In another study, P2X7R was shown to induce zeiosis to promote osteogenic differentiation and mineralization of BMSCs in postmenopausal women [65]. The authors found that BzATP (100 μM) induced the activation of protein kinase C (PKC) and Rho-related kinase, as well as cytoskeletal rearrangement in BMSCs. Basal alkaline phosphatase activity (ALP) of BMSCs was significantly delayed in postmenopausal women compared to younger women, and the results suggest that the osteogenic capacity of aging BMSCs in postmenopausal women is impaired, and this can be reversed by BzATP. Activation of P2X7R by BzATP enhanced ALP activity, expression of transcription factors RUNX2 and osterix, mineralized area, and number of bone nodules in BMSCs. Collectively, these findings provided important information to disclose the role of P2X7R in promoting the differentiation of MSCs into mature osteoblasts.

#### Mature osteoblasts

P2X7R has now been verified to be expressed in human and rodent osteoblast cell lines, such as osteoblast-like cell lines, calcareous, and bone-derived primary osteoblasts, whether it has a physiological function in osteoblasts has been controversial [46, 66, 67]. It was observed that P2X7R knockdown (KO) reduced ALP activity in osteoblasts in vitro, decreased periosteal formation in long bones of P2X7R KO mice and their osteogenic capacity under mechanical loading, and these results were similarly confirmed in human osteoblasts [68, 69]. Furthermore, the marked enhancement of mineralization in human osteosarcoma cell lines when P2X7RB (a truncated P2X7R isoform) was co-expressed with the full variant P2X7RA, suggests a positive role of fully functional P2X7R in maintaining bone strength [70]. Clearly, functional P2X7R is required during osteogenesis. However, the results of some other similar studies are contrary. Activated P2X7R induced apoptosis in SaOS-2 osteoblast cell line, induced membrane blebbing in mouse calvarial osteoblasts and MC3T3-E1 osteoblasts as well as decreased bone mineralization and ALP in primary rat osteoblasts [71–73]. Interestingly, blockade or deletion of P2X7R inhibited the propagation of intercellular calcium signals between osteoblasts and osteoclasts in human bone marrow-derived cells, and fluid shear stress caused a significant reduction in extracellular signal-regulated kinase (ERK) phosphorylation in primary mouse osteoblasts [56, 74]. It is clear that conflicting evidence for the effects of activated P2X7R on osteoblast differentiation and matrix mineralization in vitro indicated that the underlying mechanisms are not clear. Researchers attributed these differences in part to P2 receptor-dependent and/or receptor-independent mechanisms via hydrolysis of extracellular nucleotides to pyrophosphate (PPi), which is known as a mineralization inhibitor. The breakdown of ATP by ectonucleotidases can cause high-level PPi [69, 75].

In general, constitutive ATP in osteoblasts is released at a low level (approximately 0.5–1 nmol/mL) in a normal physiological environment [76]. ATP release triggered by mechanical stimulation enhances P2X7R-mediated osteogenic function, and agonist-mediated transient activation of P2X7R promotes osteoblast differentiation and matrix mineralization [77, 78]. Activation of P2X7R is involved in downstream LPA synthesis/release, PGE2 synthesis/release, and ERK1/2 activation in osteoblasts, thereby enhancing osteoblast differentiation and bone formation [38, 63, 67]. Yet, high concentrations of ATP(above 1 mM)partially inhibit bone formation, especially mineralization [79]. Such high concentrations of ATP may only occur with cellular damage (including bone tissue microdamage) or macroscopic fractures in vivo [28]. Thus, activation of P2X7R by ATP in bone may be a warning of danger in tissue or cellular damage.

#### Osteocytes

Osteocytes, the mechanosensors of bone and the primary regulator of bone homeostasis, are terminally differentiated osteoblasts. In the adult skeleton, the proportion of osteocytes reaches 90–95%, approximately 20 times that of osteoblasts. Unlike osteoblasts and osteoclasts, which are located on the bone surface, osteocytes grow in the bone matrix inside the bone, making it difficult to study them [80]. Despite this, the expression and function of P2X7R in 13 have caught attention. Evidence suggests that BzATP and liquid shear stress can induce MLO-Y4 osteocytes pore formation via P2X7R and lead to the release of PGE2, which is normally involved in the activation of downstream signals for mechanically induced bone formation [81–83]. However, conflicting evidence indicates that shear stress can also induce the release of PGE2 from MLO-Y4 osteocytes when P2X7R was inhibited [84]. It has been shown that P2X7R is important for the normal anabolic response to physical stimulation of the skeleton, as mechanical stimulation causes the release of large amounts of ATP from osteocytes, and therefore it can be hypothesized that activated P2X7R has a key role in regulating the mechanical load of osteocytes [81]. However, how it is involved in the mechanotransduction cascade of osteocytes remains unclear.

## P2X7R as the link between the immune system and osteoporosis

### P2X7R and chronic inflammatory osteoporosis

Both autoimmune and other chronic inflammatory diseases are often complicated by osteoporosis and share similar mechanisms. As mentioned previously, P2X7R is a key factor in the inflammatory and many P2X7R-coupled pathways are critical in both the inflammatory response and the regulation of bone metabolism.

**Fig. 1:**
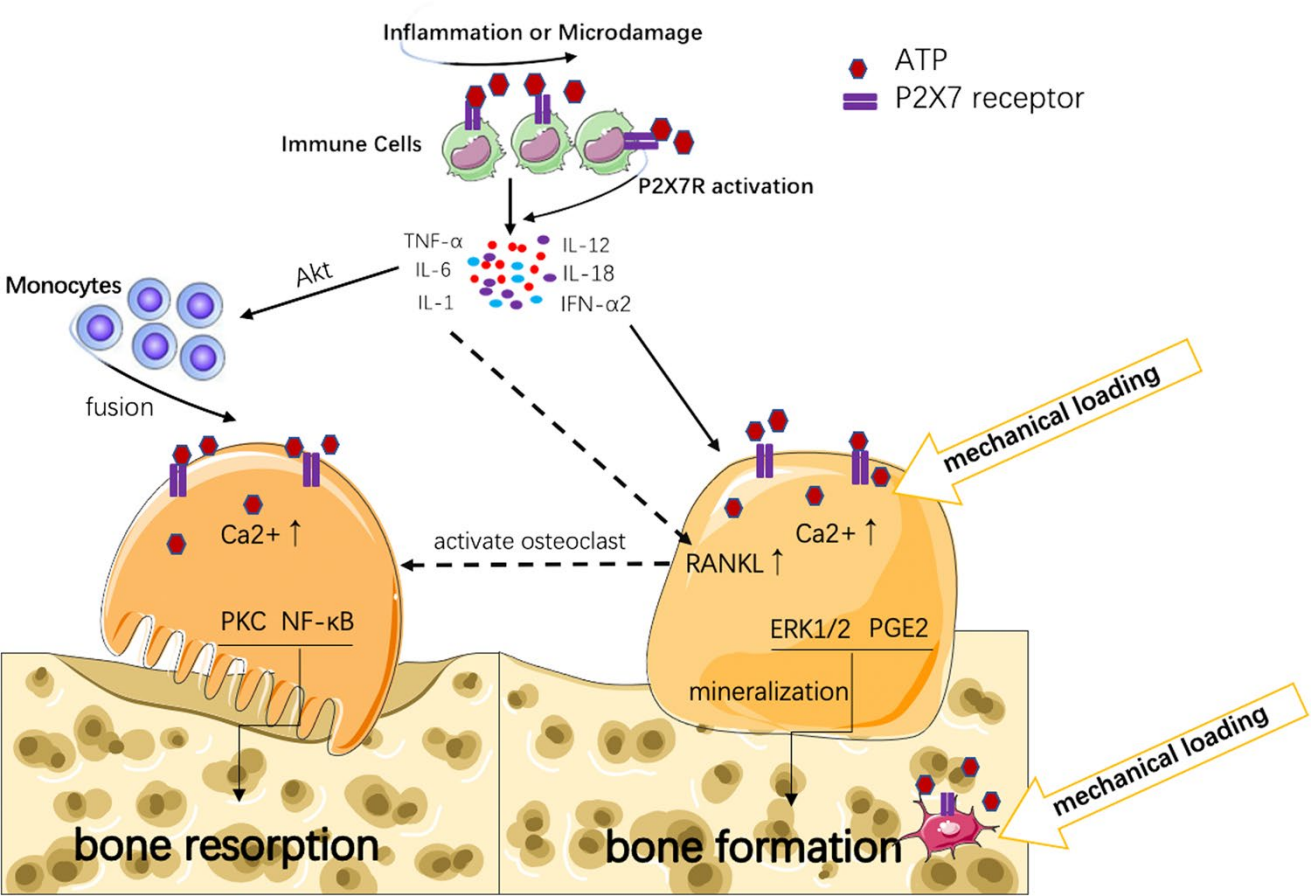
The role of P2X7R in chronic inflammatory osteoporosis. Microdamage in bone tissue or inflammation causes large amounts of ATP from immune cells which activate P2X7R, and P2X7R further exacerbates the release of inflammatory cytokines from immune cells. Inflammatory cytokines (such as IL-1, IL-6, and TNF-α) promote the fusion of monocytes to osteoclasts through AKT pathway, and enhance RANKL expression in osteoblast. RANKL promotes osteoclast formation and accelerates bone resorption. Some inflammatory cytokines (such as IL-12, IL-18, IL-33) can inhibit osteoclasts and promote osteoblast differentiation, thereby inhibiting bone loss. Mechanical stimulation can trigger the release of low-level ATP from osteoblasts or osteocytes to promote bone formation

The release of large amounts of ATP during inflammation can activate P2X7R on immune cells and the activated P2X7R further further promotes the release of inflammatory cytokines, such as IL-6 from mast cells, TNF-α from dendritic cells, and PGE2 from macrophages, ultimately maintaining and exacerbating the inflammatory [85, 86]. Persistent inflammation promotes the release of multiple cytokines such as RANKL, IL-1, IL-6, and TNF-α from immune cells. Activated P2X7R also leads to intracellular K^+^ efflux and Pannexin-1 activation, which accelerates the assembly of NLRP3 inflammasome [87, 88]. Inflammation activates caspase-1 precursors to active IL-1β by producing IL-1β converting enzyme. IL-1β inhibits osteoblast bone formation by activating the NF-kB signaling pathway, and also promotes osteoclast bone resorption by synergistic effects with TNF-α. In addition, TNF-α affects the regulation of intracellular Ca^2+^ by stimulating Akt signaling to upregulate P2 receptors, which promotes osteoclast differentiation and enhances bone resorption and accelerates the osteoporosis process [16]. Moreover, as part of the inflammatory process, the release of ATP can locally activate osteoclasts because the immune system and bone are in close contact with the bone marrow. Here, activation of P2X7R in osteoclasts can directly activate osteoclast precursors to form mature multinucleate bone resorbing osteoclasts or indirectly activate osteoclasts by stimulating P2X7R in osteoblasts and upregulating osteoblast RANKL, thereby inducing osteoclast formation [28]. However, besides the cytokines that trigger osteoclast activation (IL-1, IL-6 and TNF-α) in inflammation, cytokines that inhibit osteoclast differentiation, such as IL-12, IL-18, IL-33 and interferon alpha 2 (IFN-α2), are also present, which can inhibit bone loss [46, 89]. Thus, the composition of cytokines in inflammation is decisive for whether inflammation triggers bone loss. Moreover, the skeletal effects of the disease are further exacerbated by reduced activation of P2X7R in osteoblasts due to reduced mobility in patients with primary inflammatory disease (shown in Fig. 1).

In summary, P2X7R activation leads to upregulation of osteoclast bone resorption and downregulation of osteogenesis in osteoblasts, ultimately leading to osteolysis, which may be an important cause of osteoporosis during inflammation and appears to be influenced by high or low ATP levels.

### P2X7R and osteoporosis mediated by immune system

Immunodeficiency diseases are caused by deficiencies in immune function due to underdevelopment or acquired damage to the immune system and are classified as primary or secondary [90]. The mechanism by which it causes osteoporosis is similar to that of inflammation-mediated osteoporosis, due to the sustained activation of P2X7R and osteoclast to inhibit bone formation [91]. B cells, as the sole producers of OPG, play crucial roles in the homeostasis and regulation of bone mass. OPG can be produced by co-stimulation of CD40 on the surface of B cells and CD40L on the surface of T cells [86]. In autoimmune defects, a large number of CD4 + cells are functionally impaired and reduced in number, leading to a diminished effect of this co-stimulation, resulting in reduced OPG production, increased RANKL to OPG ratio, osteoclast activation, and accelerated bone loss, increasing the potential for osteoporosis [92].

### P2X7R gene polymorphism and osteoporosis

The gene encoding P2X7R is located on chromosome 12q24 and is highly polymorphic. More than 1500 single-nucleotide polymorphisms (SNPs) are reported in the NCBI SNP database [93, 94]. Multiple single-nucleotide polymorphisms in this gene have been demonstrated to affect the function of this receptor. Studies have shown a distinct association between SNPs in the gene encoding the P2X7R and the development of osteoporosis and fracture risk [95]. Studies have shown a noticeable association between SNPs in the gene encoding the P2X7 receptor and the development of osteoporosis and fracture risk [40, 96]. Loss-of-function polymorphisms in the P2X7 receptor gene may increase the risk of osteoporosis in postmenopausal women, and in a group of Dutch fracture patients, P2X7 receptor polymorphisms were associated with bone mineral density and risk of osteoporosis [97–99]. Genetic abnormalities in P2X7R function lead to reducing BMD and increased risk of osteoporosis [80]. Rs3751143 is widely regarded as associated with the incidence of osteoporosis [92]. It has been reported to be associated with impaired ATP-mediated pore-forming activity, with dominant pure heterozygotes having the strongest pore-forming activity, heterozygotes having the lowest pore-forming activity, and recessive pure heterozygotes losing this function altogether. Rs3751143 impairs the opening of ATP-induced cation-selective channels. Based on studies, RS3751143-C is significantly associated with the development of osteoporosis because carriers carrying the C allele have impaired ATP-induced apoptosis in osteoclasts [100–102].

Recently, it was found that P2X4R could co-expresse with P2X7R, and a heterotrophic channel could be formed between the P2X4R subunit and the P2X7R subunit. If this is true in vivo, the P2X4R subunits could theoretically replace the “defective” subunits in P2X7R to reduce the effect of genetic defects in the gene encoding P2X7R. Additionally, genes encoding P2X4R are located near and downstream of P2X7R on the same chromosome, and the polymorphism of the gene encoding P2X7R may be related to the polymorphism of the gene encoding P2X4R. So, this association could also serve as the result of gain of function or loss of function in the genes encoding P2X4R. Hence, it seems essential to investigate the association of P2X4 polymorphisms with bone status and the interaction between P2X4R and P2X7R polymorphisms [74, 103].

## The potential therapeutic targets of P2X7R for osteoporosis

P2X7R is increasingly recognized as a promising therapeutic target associated with inflammatory diseases, and a number of P2X7R inhibitors have been declared to be applie in clinical trials [104]. Many P2X7R antagonists are also used for the treatment of osteoporosis, such as A-438079 for animal studies and A740003, Ly294002, OPG, and BBG for in vitro studies [16, 105, 106]. Unfortunately, these studies have generally shown disappointing results in terms of overall disease control [107]. Besides P2X7R antagonists, some natural pharmaceutical ingredients have been shown or speculated to have positive effects in the treatment of osteoporosis by affecting P2X7R.

Puerarin, the main component of Pueraria lobata, is an isoflavone. Puerarin has been used in the clinical treatment of cardiovascular diseases and cerebral hemorrhage in China. The results showed that the neuroprotective effects induced by Puerarin were related to the control of inflammation. Nociceptive transmission mediated by P2X3R and P2X2R in primary afferent nerve could be antagonized. Puerarin also inhibited ATP-dependent IL-1 release and maturation by suppressing the expression of P2X7R protein and mRNA. Although, this result was obtained in a burn study, since the mechanism of pain and inflammation is similar to that of inflammatory osteoporosis. So, it can be inferred that geranium could also be utilized as a new drug for the treatment of osteoporosis caused by inflammation or associated with local inflammation in the future [108].

Tanshinone II-A sulfonate (TIIAS), the main component of Salvia miltiorrhiza, can completely block P2X7R-mediated Ca^2+^ influx at low molar concentrations, thereby altering ionic currents, and can treat some P2X7R-related diseases. TIIAS are non-competitive inhibitors that interfere with ATP-induced gating when they bind to intracellular structures of the receptor. Currently, Ca^2+^-activated potassium channels and phosphatase 2 are two known targets of TIIAS. TIIAS can inhibit P2X7R by acting on these two targets which in turn greatly inhibit ATP-induced Ca^2+^ influx and Yo-Pro-1 uptake, and counteracts the release and activation of the pro-inflammatory cytokine IL-1 in macrophages. Thus, TIIAS can attenuate the inflammatory response to some extent and inhibit inflammation-associated osteoporosis. Epimedoside is one of the main components of Epimedium, which was originally used in China to treat reproductive dysfunction. However, recent studies have shown that it is an osteogenic potential compound for bone repair and bone formation. Epimedoside has potent chondroprotective effects and may prevent bone degeneration in arthritis by preventing chondrocyte destruction. The results suggest that epimedoside can induce chondrogenesis by promoting gene expression and extracellular matrix synthesis in chondrocytes. Epimedoside could upregulate P2X7R gene expression, thereby inhibiting bone resorption, stimulating osteoblast differentiation, and increasing mineralization [109]. Besides pharmacotherapy, exercise therapy is gaining attention in the treatment of osteoporosis, and its mechanism of action may be related to involvement of P2X7R in skeletal mechanical signal transduction [110]. Previous studies have shown that mechanical force plays a crucial role in maintaining bone metabolic homeostasis and remodeling. Appropriate mechanical loading can effectively promote normal bone metabolism and maintain stability and health of bone structure and function. Osteoblasts, as the receptor cells of mechanical forces, stimulated by certain mechanical forces will release ATP extracellularly, which will increase the extracellular ATP concentration and stimulate the activation of P2X7R on the surface of osteoblasts and osteoclasts to regulate the bone metabolic process[111, 112]. It was demonstrated that fluid shear stress increases the secretion of PGE2 from osteoblasts and osteocytes in vitro, and that PGE2 has a major anabolic effect on bone formation but not P2X7^−/−^cells [65, 77]. P2X7R mediates ERK1/2 activation via fluid shear stress [113]. Activation of P2X7R can lead to increasing bone strength through fluid shear stress-mediated ERK1/2 activation, stimulation leading to nucleotide release and thus activation of P2X7R-mediated apoptosis in osteoclasts. P2X7R is associated with the Wnt/b-catenin signaling pathway and is involved in mechanical stress-induced bone formation in anabolic responses through increased osteogenesis in load-bearing bone in response to fluid shear stress, and has a central role in the mechanical load-induced bone formation and bone scab remodeling [66, 94]. Knockdown of P2X7R results in reduced imposed growth of long bones and cranial sutures, insufficient periosteal bone formation and trabecular bone and excessive trabecular bone resorption, resulting in reduced sensitivity of the bone to mechanical loading [88, 114]. Studies of two separate populations (210 active duty Israeli soldiers and 518 UK and American elite athletes) found that the risk of stress fracture injury was associated with a loss-of-function single-nucleotide polymorphism (rs3751143, Glu496Ala) in the gene encoding the P2X7 receptor. A P2X7R gain-of-function SNP (rs1718119, Ala348Thr) was associated with decreased incidence of sexual fracture injury [115, 116]. These evidences suggested that P2X7R mitigate osteoporosis and reduce fracture risk by promoting osteogenesis and mineralization through mechanical transduction of signals to the skeleton.

## Summary

P2X7R, as an important signaling molecule, is both a key molecule in activating the innate immune response and central to the regulation of bone metabolism. ATP-mediated purinergic signaling is probably the key to understanding inflammation-induced bone loss. Although many studies have been conducted on P2X7R-mediated regulation of bone metabolism, the full functional role of P2X7R in osteoblasts has not been fully elucidated and has been controversial, and further elucidation of the association between P2X7R function and inflammatory bone loss is therefore needed. However, there is little doubt that P2X7R is an emerging and important therapeutic target for osteoporosis, especially for exercise therapy. Whether it is possible to improve inflammation in vivo through exercise and thus improve the outcome of osteoporosis treatment needs to be further explored.

## Data Availability

Data sharing is not applicable to this article as no new data were created or analyzed in this study.

## Abbreviations

ATP: Adenosine triphosphate
P2X7R: P2X7 receptor
PMOP: Postmenopausal osteoporosis
HSCs: Hematopoietic stem cells
BMSCs: Bone marrow mesenchymal stem cells
RANKL: Nuclear transcription factor-κB receptor activator ligand
IFN-α2: Interferon alpha 2
ERK: Extracellular signal-regulated kinase
PKC: Protein kinase C
NFATc1: Nuclear factor of activated T cells 1
TNF-α: Tumor necrosis factor-α
ALP: Alkaline phosphatase activity
SNPs: Single-nucleotide polymorphisms
